# Mechanical Periodontal Therapy Recovered the Phagocytic Function of Monocytes in Periodontitis

**DOI:** 10.1155/2020/8636795

**Published:** 2020-02-15

**Authors:** Priscilla F. Naiff, Valéria M. A. Carneiro, Maria do Carmo M. Guimarães, Ana Cristina B. Bezerra, Mariangela S. Oliveira, Shirley C. P. Couto, Érica Alessandra R. Alves, Selma A. S. Kückelhaus, Maria Imaculada Muniz-Junqueira

**Affiliations:** ^1^Faculty of Health Sciences, University of Brasilia, 70910-900 Distrito Federal, Brasilia, Brazil; ^2^Periodontics' Division, University of Brasilia, 70910-900 Distrito Federal, Brasilia, Brazil; ^3^Pediatric Dentistry Division, University of Brasilia, 70910-900 Distrito Federal, Brasilia, Brazil; ^4^Laboratory of Cellular Immunology, Faculty of Medicine, University of Brasilia, 70910-900 Distrito Federal, Brasilia, Brazil; ^5^René Rachou Institute, 30190-009 Minas Gerais, Belo Horizonte, Brazil; ^6^Laboratory of Histological Techniques, Faculty of Medicine, University of Brasilia, 70910-900 Distrito Federal, Brasilia, Brazil

## Abstract

**Background:**

Several studies have focused on the association between periodontitis and systemic implications; however, the biological mechanisms of the immune responses before and after periodontal therapy involved in this relationship, such as phagocytic functions, remain unclear.

**Objectives:**

This study aimed to investigate whether periodontal treatment improves the phagocytic function of blood monocytes in patients with severe periodontitis. *Materials and Methods*. A nonrandomized sample of 55 participants was enrolled in the study. Two groups were studied: control (*n* = 27, healthy subjects without periodontal disease) and patients (*n* = 27, healthy subjects without periodontal disease) and patients (

**Results:**

Periodontitis induced impaired phagocytosis by monocytes. Phagocytosis at baseline was significantly lower in periodontitis patients [median, 13.2 (range of 7.1 to 20.8) and 60.7 (40.6 to 88.6)] than in controls [27.4 (15.5 to 40.5)] and 98 (68.2 to 122.9)] for nonsensitized or sensitized samples, respectively. After supportive therapy, patients showed a significant enhancement of phagocytic functions [33.7 (14.6 to 53.2) and 108.5 (99.6 to 159.5)] for nonsensitized and sensitized samples, respectively. Periodontal treatment increased the phagocytic capacity to a level similar to that observed in the control group and improved the capacity of phagocytes to produce superoxide anion.

**Conclusions:**

The results suggest that periodontal therapy in patients with severe periodontitis provides a state of homeostasis due to the reestablishment of phagocytic function and increased production of NBT (Regional Registry No. RBR-24T799; Universal Registry No. U1111-1133-5512).

## 1. Introduction

Periodontitis is an oral inflammatory disease triggered by the individual's immune response to microorganisms in the dental biofilm that can cause tooth loss through the destruction of periodontal tissues [1]. Considering the multifactorial aspects of biofilm and the intrinsic aspects of the affected individuals, periodontitis consists of progressive destruction of the dental insertion apparatus, where clinical loss of tooth insertion is the determining characteristic [2, 3]. In addition, bacterial virulence and inflammatory mediators resulting from parasite-host interaction may create and maintain a chronic systemic inflammatory response [1].

Periodontitis is classified according to its severity in stages, I to IV, based on the extent of interproximal tooth loss and/or the percentage of radiographic bone loss. In addition to severity, the disease is graded according to its progression (slow, moderate, or rapid) and its effects on the systemic health of individuals [4, 5].

Based on its direct relationship with systemic diseases, periodontitis is a risk factor for cardiovascular diseases and may exacerbate the complications of diabetes and those of some systemic disorders such as those of genetic, inflammatory, metabolic, and endocrine origins, which can directly affect the loss of periodontal tissues [5]. Possible pathways involved in this process include persistent, low-grade systemic inflammation caused by disseminated proinflammatory mediators and periodontium-originating bacteremia [6].

Although the process leading to chronic periodontitis is initially associated with the bacterial biofilm, tissue destruction mainly occurs due to the individual's exacerbated immune response. Among the cells of the immune system that act in this process, monocytes/macrophages produce and secrete elevated levels of metalloproteinases, reactive oxygen species (ROS), tumor necrosis factor (TNF), interleukin-1 (IL-1), interleukin-6 (IL-6), and nuclear factor kappa-Β ligand (RANK-L), which amplify the inflammatory response to control bacterial growth while leading to the destruction of periodontal tissues [7–10].

The conventional treatment for periodontitis is based on the mechanical removal of plaque and hard mineralized deposits on the surface of the teeth with the aid of mechanical tools. However, when the biofilm is deep in periodontal sites, it is possible to use access or regenerative surgeries as well as antibiotics to recover the periodontal tissues. The mechanical approach favors not only elimination of the bacterial biofilm [11, 12] but also reduction of oxidative stress and serum levels of C-reactive protein (CRP), TNF-*α*, IL-1, and IL-6 [13], resulting in reduced immunopathogenesis in the periodontium.

Considering that tooth plaque and hard mineralized deposits provide a microenvironment that stimulates production of molecules, such as TNF-a, IL-1, and IL-6, that may decrease or increase innate immune system functions, influencing the evolution of periodontitis [13] and probably systemic diseases [13], we hypothesized that mechanical periodontal treatment could normalize deregulated phagocyte functions.

With the importance of monocyte/macrophage effector mechanisms in infection control, this study aimed to evaluate the impact of periodontal disease on the phagocytic functions and superoxide anion production by cells obtained from individuals with periodontitis before and after treatment by mechanical removal of bacterial plaque. The understanding of the monocyte/macrophage functions in periodontitis may clarify the effect of mechanical therapy on cells of the innate immune system and improve the understanding of the mechanism of action of this disease and its nonsurgical therapy.

## 2. Material and Methods

### 2.1. Type of Study and Inclusion and Exclusion Criteria

This is a descriptive, controlled, paired, comparative, and nonblinded experimental clinical study to determine the effect of mechanical periodontal therapy on the function of monocytes/macrophages obtained from peripheral blood of individuals with periodontitis.

The clinical study (Regional Registry No. RBR-24T799; Universal Registry No. U1111-1133-5512) was approved by the Human Research Ethics Committee of the University of Brasília (process number 0067.0.012.012-08) and was developed in agreement with Brazilian legislation and the revised Declaration of Helsinki in 2013. The study design was based on CONSORT (Consolidated Standards of Reporting Trials) recommendations [14].

The inclusion criteria for participation in this study were patients with periodontitis who had no disease other than periodontitis. The diagnosis of periodontitis was based on the current Classification of Periodontal Diseases [4, 5]. The following conditions were considered exclusion criteria: patients undergoing periodontal treatment and antimicrobial agent usage in the last 12 months; therapies with anti-inflammatories, corticosteroids, or immunosuppressant agents; smokers for less than 5 years; pregnant or lactating women; oncologic and diabetic patients; autoimmune, infectious, or allergic conditions; and morbidly obese (BMI > 40 kg/m2) or malnourished (BMI < 1.8.5 kg/m2) individuals.

All subjects agreed to participate in the study by signing the informed consent form.

### 2.2. Study Group

Initially, volunteers were interviewed to collect epidemiological data (age, sex, and smoking habits), and they underwent clinical examination and mechanical periodontal therapy by a single experienced examiner (VMAC) in the Periodontal Clinic at University Hospital of Brasília (HUB), Federal District, Brazil.

Clinically, probing depth (PD), clinical attachment level (CAL), visible plaque index (PI) [15], and gingival bleeding on probing (BOP) [16] were evaluated with a periodontal probe (Michigan O with Williams marking) at four sites on each tooth (buccal, mesial, distal, and palatine/lingual), with the exception of the third molar teeth. The greatest depth of periodontal site was recorded on the proximal mesial and distal surfaces. These same clinical parameters were reevaluated at the end of periodontal therapy, considering the remission of clinical signs of inflammation, such as absence of BOP and residual periodontal pockets.

The calibration and measurements of PD and CAL were repeated within 24 hours and demonstrated agreement of over 80%. BOP was calculated by the Kappa coefficients, and the intraexaminer agreement was >0.85.

**Figure 1:**
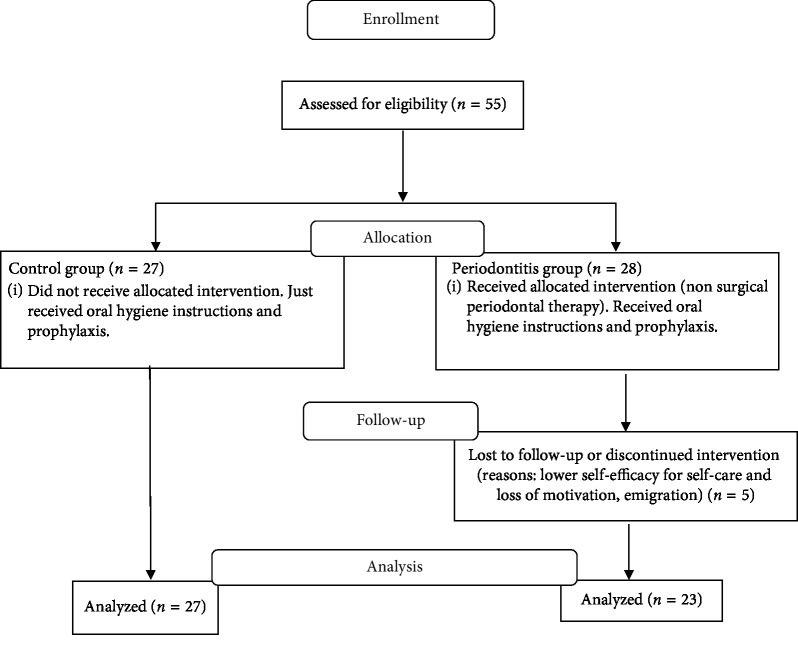
The flowchart of the study before and after periodontal therapy according to the consolidated standards of Reporting trials—CONSORT.

From the clinical exams, 55 study participants (18 men and 37 women) were grouped. Thus, the control group consisted of 27 individuals (21 to 44 years) with no evidence of periodontitis, without radiographic evidence of bone loss (BOP <10% sites, buccal CAL <3 mm, and absence of interproximal CAL and PD ≤ 3 mm) and with a minimum of 20 preserved teeth. The intervention group consisted of 28 individuals (20 to 45 years old), BOP > 10% sites, clinical (PD ≥ 4 mm, CAL ≥ 3 mm), and radiographic diagnosis of periodontitis in 18 or more teeth in stages II, III, and IV, according to Armitage [17], Armitage and Cullinan [18], and Caton et al. [4]. Five individuals from this group did not follow the treatment, which resulted in 23 individuals for the paired study (Figure 1).

### 2.3. Treatment Protocol

During the study, the subjects in the intervention group underwent mechanical removal of plaque without antibiotic administration in three stages: (a) in the first stage, mechanical periodontal therapy occurred within 14 days of the study, using supragingival and subgingival instrumentation (scaling and root planing) with Gracey's curettes (Millenium Golgran, BR) to remove biofilm and dental calculus; (b) the second stage, which occurred between 30 and 180 days after the beginning of the study, was reinstrumentation of the periodontal sites with persistent deep pockets, bleeding on probing, and calculus. At this stage, meticulous scaling was performed to reduce the number of sites with a depth of 4 or 5 mm, respectively, to 3 or 2 sites, as well as to reduce the plaque index (≤15%) and bleeding index (≤10%); and (c) the third stage of periodontal therapy consisted of supporting patients for plaque control through meticulous professional dental cleaning and reinforcement of patients' oral hygiene procedures. In this phase, participants were followed up with every 15 or 30 days for six months.

All participants of the study received oral self-hygiene instructions for correct interdental cleaning and teeth brushing. At each dental visit for periodontal evaluation and therapy, motivation and home oral health advice were reinforced to these individuals.

### 2.4. Functional Evaluation of Monocytes

Initially, peripheral blood was obtained by venipuncture from each participant (before and after periodontal treatment) in vacuum blood collection tubes without anticoagulant (Vacuteiner®, USA) to evaluate the phagocytic capacity and production of superoxide anion. Laboratory tests were performed by experienced examiners (SCPC; MSO, EARA).

#### 2.4.1. Phagocytic Capacity

For phagocytosis, a technique for leukocyte adhesion on slides was adapted from that described by Muniz-Junqueira et al. [19]. An aliquot (40 *μ*L/round fields) of whole blood was deposited on 8 demarcated round fields (7 mm diameter) on slides for microscopy and incubated in a humid chamber for 45 min at 37°C. Afterward, the slides were washed with phosphate-buffered saline (PBS) at 37°C (0.15 M, pH 7.2) to remove nonadherent cells, and a suspension of *Saccharomyces cerevisiae* (2.5 × 10^5^/well) was added to 20 *μ*L of Hanks-tris pH 7.2 (Sigma, USA) in each well. The yeast was previously sensitized with 10% of the individual's own serum to evaluate phagocytosis for opsonin receptors or with 10% fetal bovine serum (FBS) previously inactivated at 56°C (Gibco, USA) to evaluate phagocytosis for pathogen-associated molecular pattern receptors [19–22]. After incubation of the phagocytes with the yeast for 30 min in a humid chamber at 37°C, the preparations were washed with PBS, fixed with methanol, and stained with 10% Giemsa solution. The phagocytic index was calculated by the product of the average number of phagocytosed yeast per phagocytosing monocyte by the percentage of monocytes involved in phagocytosis [23].

#### 2.4.2. Production of the Superoxide Anion

The nitro blue tetrazolium (NBT) salt reduction method [20, 24] was used to evaluate the production of superoxide anion. This radical oxygen species reduces the compound NBT to an insoluble form, formazan, which is visualized by optic microscopy by a blue color in the cytoplasm of the phagocytes. The percentage of cells that reduced the NBT is directly proportional to the amount of oxygen radicals (superoxide anion) produced by phagocytes [24]. The phagocytes adhered on the slide, as described above, were incubated with 0.05% NBT solution in Hanks-tris (Sigma, USA) for 20 min in a humid chamber at 37°C (basal yield). Stimulated superoxide anion production was evaluated after a suspension of *S. cerevisiae* was added at a ratio of 1 cell/5 yeast per well. The slides were then washed, fixed with methanol, and stained with a solution of 1.4% safranin and 28.6% glycerol in distilled water. The results are given by the percentage of phagocytes (monocytes/macrophages + neutrophils) that reduced the NBT salt as analyzed by optical microscopy.

### 2.5. Statistical Analysis

The sample size was determined for a desired power of 90% and an alpha level of significance of 0.05 to be a minimum number of 25 individuals by group using Sigma Stat software. The results were evaluated using Bartlett's test for equal variances and the Kolmogorov–Smirnov test for normal distribution before comparative analysis. Considering the unrelated samples, a *t*-test or Mann–Whitney test were used to compare two groups with normal or nonnormal distribution, respectively. Two dependent samples were compared with a paired *t*-test or Wilcoxon test for normal or nonnormal distribution, respectively. The Prism 5.0 software package (GraphPad, USA) was used for statistical tests and graphical presentation of the data; differences with a two-tailed value of *p* < 0.05 were considered statistically significant.

## 3. Results

### 3.1. Clinical and Demographic Aspects

Periodontal therapy and the patient maintenance period were completed between 9 and 12 months after the start of the study based on the individual oral hygiene procedures and the healing of periodontal tissues. Thus, 82% (*n* = 23) of patients completed the periodontal treatment and the follow-up period proposed in the study (Figure 1). The study diagram and the epidemiological profile of individuals are shown in Figure 1 and Table 1.

Our results showed that individuals with periodontitis had a higher percentage and number of sites with gingival bleeding with biofilm presence and clinical attachment level (CAL) than the control group at the beginning of the study but not at the end of supportive therapy, in which the control and intervention groups showed similar results (Table 2).

### 3.2. Evaluation of Phagocytic Capacity

**Figure 2:**
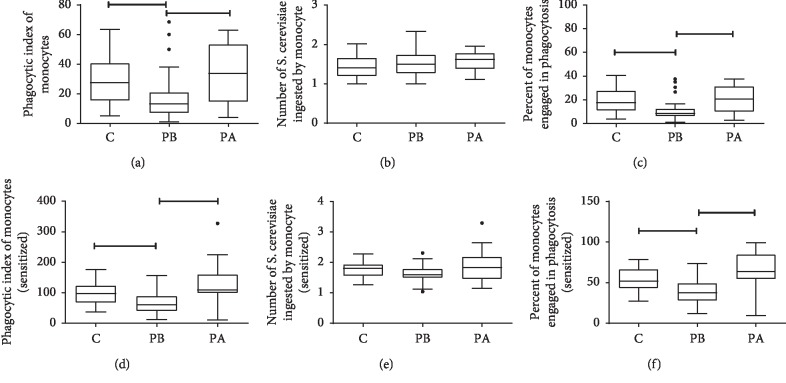
Phagocytic capacity of monocytes/macrophages obtained from peripheral blood of control subjects (C *n* = 27) or with periodontitis (P *n* = 28) before (PB) and after (PA) the mechanical removal of plaque. The cells were incubated with *S. cerevisiae* sensitized with fresh human serum (opsonin phagocytosis) or with inactivated FBS (phagocytosis for pathogen-associated molecular patterns) to determine the phagocytic index (PhI) (a, d), which is the product of the phagocytosed yeasts/cell mean (b, e) by the % of cells involved in phagocytosis (c, f). Before therapy, the results showed lower PhI in the periodontitis group than in the control group for both phagocytosis by opsonins and by pathogen-associated molecular patterns (Mann–Whitney, *p* < 0.05); the paired analysis showed that treatment of the individuals increased the PhI in the periodontitis group (PA > PB) by the two pathways of phagocytosis (Wilcoxon, *p* < 0.05). The medians, quartiles, and maximum and minimum values are shown.

The results showed that, before treatment, the median phagocytic index of monocytes/macrophages of individuals of the periodontitis group was lower than that of the control group in either phagocytosis in the presence of opsonins (control = 98.0; periodontitis = 60.7) or phagocytosis by pathogen-associated molecular pattern receptors (control = 27.4; periodontitis = 13.2) (Mann–Whitney, *p* < 0.05) (Figures 2(a) and 2(d)). After treatment, the periodontitis group did not differ from the control group (*p* > 0.05). The results analyzed by the Wilcoxon test show that the treatment was able to increase the median phagocytic index of monocytes/macrophages by opsonin receptors (before = 60.7, after = 108.5) (*p* < 0.05) or by pathogen-associated molecular pattern receptors (before = 13.2, after = 33.7) (*p* < 0.05) by the increase in the percentage of cells involved in phagocytosis (Figures 2(c) and 2(f)).

### 3.3. Evaluation of Superoxide Anion Production

The evaluation of superoxide anion production showed that the percentage of nonstimulated NBT reduction in the control group (*C* = 76.5%) did not differ from the periodontitis group before starting treatment (PB = 75.5%) (Mann–Whitney, *p* > 0.05); similarly, the same was observed for the % reduction of NBT stimulated with *S. cerevisiae* (*C* = 69.5%; PB = 73%) (Mann–Whitney, *p* > 0.05) at baseline. After treatment, the percentage of nonstimulated NBT reduction (PA = 83.5%) or stimulated (PA = 79%) was higher than that for the control group (*C* = 76.5% nonstimulated, 69.5% stimulated) (Mann–Whitney, *p* < 0.05).

**Figure 3:**
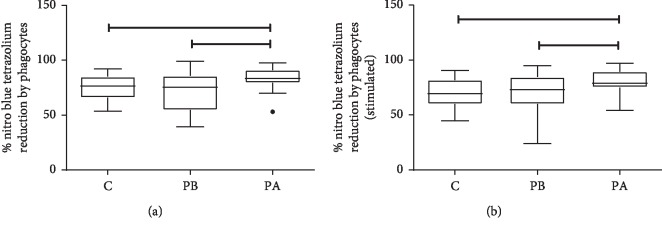
Percentage of NBT reduction, nonstimulated (a) or stimulated (b), by cells obtained from control individuals (C) or with periodontitis before (PB) or after (PA) mechanical treatment for plaque removal. The results showed that the treatment increased the percentage of nonstimulated and stimulated NBT reduction compared with the control group (PA > C) (Mann–Whitney, *p* < 0.05) or with the cells obtained before treatment (PA > PB) (Wilcoxon, *p* < 0.05). There were no differences in the percentage of NBT reduction by control group cells and those obtained from periodontitis before treatment (PB) (Mann–Whitney, *p* > 0.05).

The paired study showed that the cells obtained from the periodontitis group after treatment increased the percentage of reduction of the nonstimulated NBT (PA = 83.5%) (Figure 3(a)) or stimulated (PA = 79%) (Figure 3(b)) when compared with the time before treatment (nonstimulated PB = 75.5%, stimulated PB = 73%) (Wilcoxon, *p* < 0.05).

## 4. Discussion

This is the first prospective intervention study that evaluated the effect of mechanical periodontal treatment in phagocytosis by monocytes. Furthermore, we also showed that this treatment improved the capacity of phagocytic cells to produce superoxide anion in patients with periodontitis.

Poor oral hygiene increases the risk of oral and systemic diseases [1, 15], so it is very important to motivate people to perform oral self-care activities, such as frequent teeth brushing and interdental cleaning, at home. The reduction of adverse outcomes along with improved quality and even quantity of life is worth the effort in changing hygiene habits. Our study design enabled scheduled dental appointments to clinically evaluate the oral health improvements achieved by patients individually as a result of both periodontal mechanical treatment and the oral hygiene habits of each person who participated in the study.

In the clinical protocol of the present study, the time after periodontal therapy for the reassessment of the final clinical and phagocytic parameters was not the same because patients achieved complete resolution of the inflammation at different times due to the individual characteristics and responses among the immunological systems of each person. Each patient was frequently examined, and reinstrumentation of periodontal sites was made according to the needs of each patient until complete resolution of the inflammation was reached, as indicated by the absence of bleeding on probing and residual pockets when the periodontal therapy was considered complete. Consequently, the reassessment of the clinical parameters and new peripheral blood collection for monocyte phagocytosis and NBT testing were performed according to the resolution of each patient. This period covered nine to twelve months. The rigorous clinical protocol used in this work allowed all patients to have a similar level of clinical resolution of inflammation at the end of supportive therapy.

In our study, participants with the former aggressive (AgP) and chronic periodontitis (CP) clinical forms were grouped in a single group (periodontitis group), as suggested by the new classification of periodontitis [4]. This classification was based on the fact that both forms share the same immunopathological pathways and consequently should present similar performance by cells of the innate immune system [21].

Before treatment, phagocytosis by monocytes of patients with periodontitis was less than that of healthy individuals. Furthermore, our data showed that the deficiency in phagocytosis before treatment was more evident when phagocytosis was assessed through complement and antibody receptors (sensitized). Our results corroborate the findings of another study that showed impaired phagocytosis by monocytes from peripheral blood in AgP [25]. The authors suggested that the impairment in phagocytosis of opsonized zymosan might be attributed to a genetic factor, such as polymorphism of Fc*γ* receptors on leukocytes of periodontal disease patients, which would predispose them to less efficient phagocytosis. We also hypothesize that the observed decrease in the phagocytosis of monocytes before therapy may be due to the high systemic level of proinflammatory cytokines produced during the host-pathogen interaction. These regulatory factors in the serum may downregulate the function of neutrophils and monocytes [25–27].

Mechanical treatment resulted in an increase in the phagocytic index. The monocytes from patients presented improved phagocytic function after therapy. Healing of periodontal tissues and the resolution of inflammation after treatment in the patients with impaired phagocytosis may have led to the withdrawal of factors that were suppressing the cell functions, which allowed recovery of the phagocytic function.

This increase in PhI was caused by an increase in the proportion of cells engaged in phagocytosis because therapy did not influence the average number of yeast ingested by monocytes. A possible explanation is that this increase could be related to tissue repair involving ratios of different monocyte lineages (M1/M2).

M1 or classical monocytes produce inflammatory mediators, and they are rapidly recruited to sites of infection and damaged tissue to provide defense against invasive microorganisms. They are stimulated by lipopolysaccharides and proinflammatory cytokines, such as IL-1*β* and IFN-*γ*. M2 or nonclassical monocytes contribute to tissue repair after injury (by stimulating angiogenesis and extracellular matrix synthesis, rich in collagen) and are mediated by anti-inflammatory cytokines secreted by macrophages, mainly IL-4, IL-10, and TGF-*β*. There is evidence that regulatory T cells are capable of promoting the polarization to M2 macrophages by the IL-10 and TGF-*β* pathways in addition to inhibiting M1 subtype induction by T-cell effectors [28]. Another study found out that when macrophages were cocultured with human gingiva-derived mesenchymal stem cells (GMSCs), they acquired the M2 phenotype [29]. The authors also demonstrated that systemically infused gingiva-derived mesenchymal stem cells could migrate to the wound site and promote M2 macrophage polarization, significantly enhancing wound repair.

An imbalance in the number of the M1 and M2 monocytes in periodontitis might explain our findings. Before therapy, it is possible that the M1 monocytes prevailed in the microinflammatory environment. After supportive mechanical treatment, which provides tissue repair and returns the periodontium to normality, M2 monocytes prevailed, and phagocytosis by these monocytes increased to remove damaged cells and debris, aiding in tissue remodeling and leading to the average proportion between M1/M2 monocytes that is found in healthy individuals. This idea agrees with the finding that both M1 and M2 phenotypes of macrophages are activated and enhanced in periodontitis, but the phenotype that prevails seem to tend toward M1 macrophages [28]. However, in this study, the presence of cell surface receptors and cytokines responsible for the onset of both monocyte populations was not evaluated, and our hypothesis needs to be confirmed in subsequent studies.

Unlike previous findings [30, 31] of higher ROS production by stimulated neutrophils in chronic periodontitis patients than in healthy volunteers, in this study, periodontal disease did not influence the capacity of production of superoxide anion compared with the control group at baseline.

Nonsurgical periodontal treatment was effective in reducing reactive oxygen metabolite (ROM) levels in another study that measured ROM by the total serum oxidant capacity against *N*,*N*-diethylparaphenylendiamine in an acidic buffer [11]. In addition, a recent study found decreased oxidative stress marker production in gingival crevicular fluid after periodontal therapy, which was not found in the serum. They concluded that initial periodontal therapy might be helpful for reducing local but not systemic oxidative stress in periodontitis [12].

As our patients showed significant clinical improvement after treatment, a possible explanation is that the increased levels of superoxide anion after treatment may have caused positive effects in tissue repair. Benefits of ROS release were found during dental treatments using low-level laser therapy (LLLT) [32]. Photostimulation by LLLT of mitochondria on human fibroblasts *in vivo* and human adipose-derived stem cells *in vitro* increase the level of ATP, with a subsequent transient increase in ROS levels. This process was related to reduction/oxidation (redox) signaling in cells that are involved in cellular homeostasis and proliferative control [33–35]. ROS activation in LLLT activates growth factors, cell proliferation, angiogenesis, and tissue repair processes [36, 37]. Periodontal therapy may act as LLLT, leading to a nonexcessive increase in ROS release. It is possible that the increase in oxygen radical production posttreatment was sufficient to cause a wound-healing response in periodontal tissues.

A possible limitation of this study is the extrapolation of an in vitro laboratory finding for a clinical condition that involves a multiplicity of interplaying functional factors. Another possible limitation of this work was that we used dead *S. cerevisiae* and not periodontal pathogens to investigate phagocytosis. When using live bacteria, their virulence factors may influence phagocytosis. Thus, when investigating the phagocytosis, two lines of reasoning can be defined: the direct action of bacteria in phagocytosis and the effects of host-parasite interactions in phagocytosis. In addition to these lines, phagocytosis could be analyzed at the site of infection/inflammation or systemically. As our aim was to evaluate the effects of this interaction in the host in monocytes from blood and not from periodontitis sites, this justifies another stimulus provided to the cell, including yeast. Furthermore, *S. cerevisiae* was chosen as the particle to be phagocytosed because it is taken up by the same receptors in monocyte/macrophages as those that phagocytose pathogenic bacteria present in periodontitis [21]. Despite these limitations, our findings reinforce the concept that periodontitis may promote significant immunological changes in cells from human peripheral blood, the systemic impacts of which need to be better understood.

Further research should be performed to investigate the immunoinflammatory events implicated in phagocytosis and ROS production by monocytes in periodontitis.

## 5. Conclusions

In conclusion, our data showed that phagocytic deficiency induced by periodontitis could be reversed through periodontal therapy, which can reestablish monocyte functions to a state of homeostasis, as observed in the control. Our data suggest that periodontal treatment could reduce the risk of systemic disorders modified or aggravated by periodontitis by improving or restoring immunologic functions, such as phagocytosis and ROS production.

## Figures and Tables

**Table 1:** Epidemiological profile of studied subjects.

Groups	Number and percentage		Age (years) mean ± SD	Number of teeth mean ± SD	Patients/endpoint
	Male	Female			
Control	9 (33%)	18 (67%)	33.2 ± 6.4	28.8 ± 2.0	—
Periodontitis	9 (32%)	19 (68%)	34.36 ± 6.2	27.3 ± 4.8	9 months (n = 10)
					10 months (n = 10)
					12 months (n = 3)

**Table 2:** Clinical parameters assessed before and after periodontal support therapy.

Clinical parameters (% of sites)		Control (*C*)	Periodontitis		Statistical analysis (*P* value)		
			Before therapy (PB)	After therapy (PA)	PB × PA	PB × C	PA × C
Plaque index		4.7 ± 2.3	63.6 ± 33.6	4.8 ± 6.7	<0.0001^1^	<0.0001^3^	=0.0687^3^

Bleeding on probing		2.6 ± 1.4	44.4 ± 29.3	1.6 ± 3.3	<0.0001^1^	<0.0001^3^	0.0687^3^

Probing depth	≤3	100.0	68.7 ± 14.3	98.3 ± 1.7	<0.0001^2^	NA	
	4	—	4.0 ± 4.0	0.6 ± 0.9	<0.0002^1^		
	5-6	—	17.0 ± 8.8	0.8 ± 1.2	<0.0001^1^		
	≥7	—	10.4 ± 8.8	0.1 ± 0.6	<0.0001^2^		
Clinical attachment level (mm)	≤3	100.0	62.5 ± 18.2	—	—		
	4	—	4.9 ± 4.7	—	—		
	5-6	—	18.7 ± 8.9	—	—		
	≥7	—	13.7 ± 11.4	—	—		

NA = not applicable. ^1^Wilcoxon test. ^2^Paired *t*-test. ^3^Mann–Whitney test.

## Data Availability

The detailed data from phagocytosis and nitro blue tetrazolium's tests used to support the findings of this study are available within the supplementary information file.

## Supplementary Materials

Supplementary Materials Table S1: detailed results from phagocytosis and nitro blue tetrazolium's tests.
