# Supplementary material for: Mechanical Periodontal Therapy Recovered the Phagocytic Function of Monocytes in Periodontitis
Source: Int J Dent. 2020 Feb 15;2020:8636795. doi: 10.1155/2020/8636795 (PMC7044476; doi:10.1155/2020/8636795)
Supplement: Supplementary Materials — Table S1: detailed results from phagocytosis and nitro blue tetrazolium's tests. [file 8636795.f1.pdf]

Supplementary Material

Table S1: Detailed results from phagocytosis and nitro blue tetrazolium's tests.

					p value		
		**C**	**PB**	**PA**	**PB X PA**	**C X PB**	**C X PA**
PhI	ns	27.4 (15.5 - 40.5)	13.2 (7.1 - 20.8)	33.7 (14.6 - 53.2)	0.005[1]	0.02[2]	0.5[2]
	s	98 (68.2 - 122.9)	60.7 (40.6 - 88.6)	108.5 (99.6 - 159.5)	0.001[1]	0.006[2]	0.06[2]
Nº ingested yeasts	ns	1.4 (1.2 - 1.6)	1.5 (1.2 - 1.7)	1.6 (1.3 - 1.7)	0.3[3]	0.2[4]	0.01[4]
	s	1.8 (1.5 - 1.9)	1.6 (1.5 - 1.8)	1.8 (1.4 - 2.1)	0.1[1]	0.1[4]	0.6[2]
% Cells in Ph	ns	17.5 (11 - 27.5)	8.5 (6 - 12.4)	20.5 (10 - 31.2)	0.003[1]	0.01[2]	0.9[2]
	s	51.7 (43.2 - 66.3)	37.2 (27.8 - 49.3)	63.5 (54.3 - 84.5)	0.0002[3]	0.003[4]	0.03[4]
NBT	ns	76.5 (65.7 - 85)	75.5 (54.7 - 85.7)	83.5 (79.2 - 91)	0.005[1]	0.4[2]	0.01[2]
	s	69.5 (60 - 81.7)	73 (60 - 84.5)	79 (75 - 89.5)	0.01[3]	0.6[4]	0.003[4]

C = Control group, PB = Periodontitis group before therapy, PA = Periodontitis group after therapy, PhI = Phagocytic Index, % Cells in Ph = Proportion of cells involved in phagocytosis, NBT = Nitroblue tetrazolium test, ns = nonsensitized *S. Cerevisiae*, s = sensitized *S. Cerevisiae*, stim = stimulated; [1]Wilcoxon test, [2]Mann Whitney test, [3]Paired t test, [4]t test. Results were expressed as median (lower - upper quartiles).
